# A case of insulinoma effectively treated with low‐dose diazoxide

**DOI:** 10.1002/ccr3.3017

**Published:** 2020-06-03

**Authors:** Atsushi Yasuda, Toshiro Seki, Natsumi Kitajima, Tanefumi Baba, Noriko Sasaki, Yoshie Kametani, Masami Seki, Kazuko Tanaka, Masayuki Oki

**Affiliations:** ^1^ Division of Nephrology, Endocrinology and Metabolism Department of Internal Medicine Tokai University School of Medicine Kanagawa Japan; ^2^ Department of Neurosurgery Tokai University School of Medicine Kanagawa Japan; ^3^ Division of Rheumatology Department of Internal Medicine Tokai University School of Medicine Kanagawa Japan; ^4^ Department of Molecular Life Science Division of Basic Medical Science Tokai University School of Medicine Kanagawa Japan; ^5^ Seirei Numazu Hospital Shizuoka Japan; ^6^ Division of General Internal Medicine Department of Internal Medicine Tokai University School of Medicine Kanagawa Japan

**Keywords:** diazoxide, hypoglycemia, insulinoma, neuroendocrine tumor

## Abstract

Diazoxide is a benzothiadiazine that can be effective in managing hypoglycemia in frail patients with surgical risk. We report here a case of insulinoma effectively treated with diazoxide, as our report will be helpful for similar cases.

## Graphical abstract

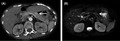

## 1 INTRODUCTION

We present a case of insulinoma effectively treated with low‐dose diazoxide. Surgical resection was considered risky in the patient with low cardiac function and history of SLE. We were able to control blood glucose by increasing the drug to the recommended lower limit.

Insulinoma is a type of neuroendocrine tumor of the pancreas that affects 1‐4 people per million each year.^1^ Although adult cases of hyperinsulinemic hypoglycemia resulting from insulin secretion from pancreatic beta cells may be caused by gastric bypass surgery or insulin receptor gene mutations, most cases are due to insulinoma.^2^

Surgery is the first choice for the treatment of insulinoma, but 5%‐10% of cases are inoperable due to malignancy or tumor recurrence.^3^ Pharmacological options and nonsurgical modalities such as radio‐frequency ablation are reserved for these cases.^4^ The pharmacological options include; somatostatin analogs, streptozotocin, mammalian target of rapamycin (mTOR) inhibitors such as everolimus, and diazoxide.^5^, ^6^, ^7^, ^8^ However, pharmacological options are not always effective in managing patients' symptoms.

Diazoxide inhibits insulin secretion and increases blood glucose by opening potassium channels of β cells.^6^ This effect is reversible and is used in the treatment of hyperinsulinemic hypoglycemia, including insulinoma. On the other hand, it can be difficult to adjust the dosage due to its wide range of adverse effects such as edema, hypotension, hirsutism, palpitation, tachycardia, nausea, vomiting, and cytopenia.^9^ The recommended starting and maintenance dose ranges are wide, and the dose should be determined according to the patient's condition. We report here a case of insulinoma effectively treated with low‐dose diazoxide, as this case report will be helpful for similar cases in the future.

## 2 CASE REPORT

The patient is a 69 years old female who was diagnosed with systemic lupus erythematosus (SLE) 5 years ago and is currently being maintained on prednisolone (PSL) 30 mg/d. She was admitted to our hospital with fever and disturbed level of consciousness and was eventually diagnosed with central nervous system lupus. PSL 40 mg/d was administered as an intravenous infusion for 2 weeks, after which she was switched to an oral dose of 40 mg/d. She was given trimethoprim‐sulfamethoxazole as a prophylaxis against opportunistic infections for a duration of one year. She was given a daily dose of 80 mg for trimethoprim (about one‐eighth of the usual dose). Later, she experienced an attack of hypoglycemia (blood glucose level = 40 mg/dL), and detailed examination was initiated in our department.

On physical examination, the patient was 150.0 cm tall and weighed 31.0 kg. The patient's vital signs were as follows: temperature, 36.5°C; pulse, 101 beats/min; blood pressure, 120/70 mm Hg; and respiratory rate, 18 breaths/min. She had mild consciousness disturbance (Glasgow Coma Scale = 14). She had conjunctival pallor and no conjunctival jaundice. The chest was clear on auscultation. Cardiac examination revealed a systolic murmur in the aortic valve area. The abdomen was not tender, and there was no skin rash or edema in either limb. Neurological examination did not reveal any abnormal findings.

**Figure 1:**
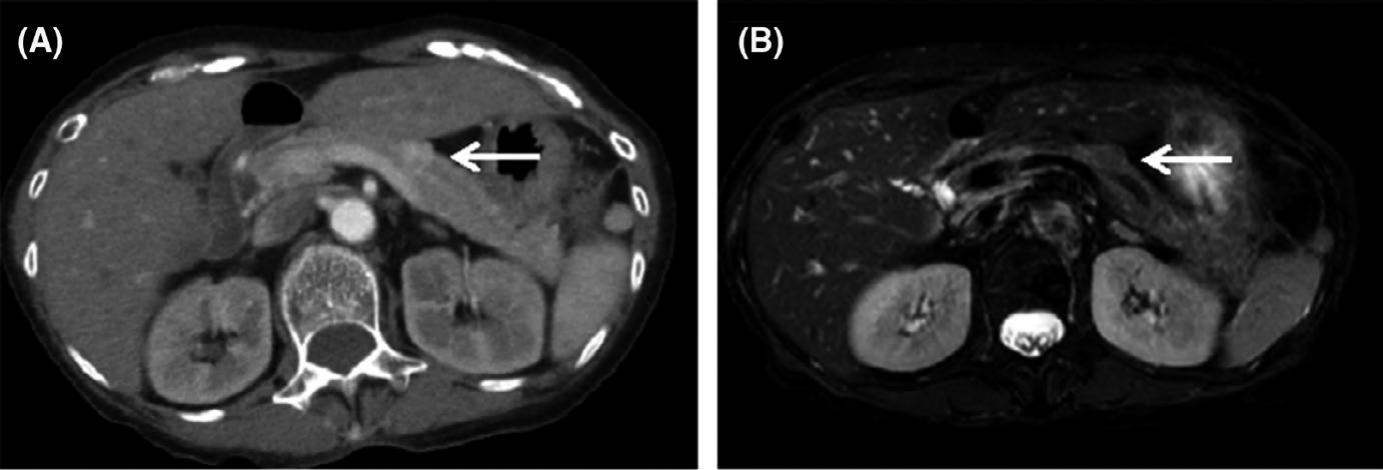
Computed tomography (CT) and magnetic resonance imaging (MRI) findings. CT (A) shows a 9‐mm‐diameter nodule (arrow) in the pancreatic body, and MRI (B) shows a low‐signal area at the same site (arrow)

**Figure 2:**
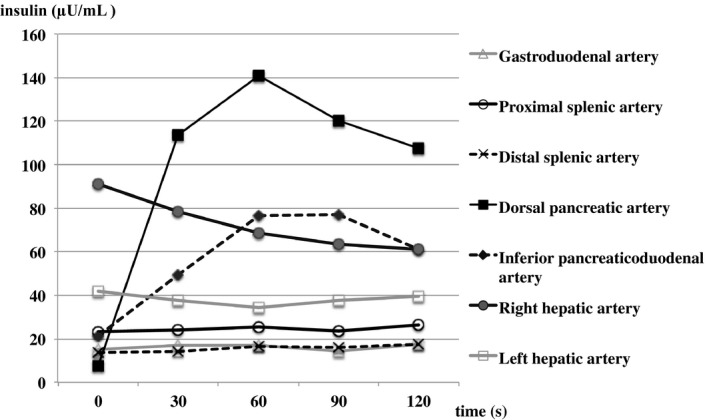
The results of the arterial stimulation and venous sampling test (ASVS). Results of ASVS showed significant elevation in insulin level in the dorsal pancreatic artery and in the lower pancreaticoduodenal artery

As shown in Table 1, blood tests showed pancytopenia, antibody abnormalities, and hypocomplementemia thought to be due to SLE. Malnutrition and hypokalemia, which seemed to be an effect of steroids, were observed. Furthermore, brain natriuretic peptide (BNP) was high, and echocardiography showed that the area of the aortic valve was 0.9 cm^2^, with severe aortic stenosis and low ejection fraction (EF) of 48%. First, we suspected hypoglycemia associated with PSL dose reduction and drug‐induced hypoglycemia due to the trimethoprim‐sulfamethoxazole combination. However, laboratory investigations showed a fasting hypoglycemia of 32 mg/dL, the serum immunoreactive insulin (IRI) of 7.7 μU/mL, and the C‐peptide of 0.88 ng/mL, all of which suggested inappropriate secretion of insulin (Table 2). Regarding the functions of the pituitary gland, thyroid gland, and adrenal gland, there were no abnormal findings that could cause hypoglycemia (Table 2). Abdominal echo showed a 12 mm clear and marginal tumor in the pancreatic body. Contrast‐enhanced computed tomography (CT) showed a tumor in the body of the pancreas, and magnetic resonance imaging (MRI) showed a low‐signal area at the same site (Figure 1). The arterial stimulation and venous sampling test (ASVS) was performed, and the dorsal pancreatic artery, which was the dominant vessel at the site where the tumor was located, and the lower pancreaticoduodenal artery, which is a branch of the dorsal pancreatic artery, reacted at more than twice the previous values after loading and had significantly increased insulin levels (Figure 2). Therefore, a diagnosis of benign insulinoma in the dorsal pancreatic artery and inferior pancreaticoduodenal artery region was made.

**Table 1:** Laboratory data before starting the treatment with diazoxide

Urinalysis		Blood chemistry			
Color	Yellow	CRP	1.05 mg/dL	Ca	8.1 mg/dL
pH	8.0	TP	5.9 g/dL	P	2.6 mg/dL
Protein	(‐)	Alb	3.1 g/dL	BUN	13 mg/dL
Occult blood	(‐)	AST	25 IU/L	Cr	0.31 mg/dL
Glucose	(‐)	ALT	19 IU/L	UA	2.9 mg/dL
Ketone bodies	(‐)	LDH	353 IU/L	glucose	28 mg/dL
		ALP	173 IU/L	HbA1c	4.8%
		γ‐GTP	12 IU/L	BNP	253.0 pg/mL
Hematological investigations		CPK	25 IU/L		
WBC	2100/µL	Na	142 mEq/L		
Neu	86.0%	K	2.9 mEq/L		
Lym	6.0%	Cl	106 mEq/L		
Mo	6.5%	Immunological investigations			
Eo	0.5%	Antinuclear antibodies	positive		
Ba	0.5%	Anti‐dsDNA antibodies	106 U/mL		
RBC	278 × 10^4^/µL	Anti‐SS‐A Antibodies	4 U/mL		
Hb	8.7 g/dL	Anti‐insulin antibody	<0.4 U/mL		
Ht	29.8%				
Plts	13.6 × 10^4^/µL				

Abbreviations: Alb, albumin; ALP, alkaline phosphatase; ALT, alanine aminotransferase; Anti‐dsDNA, antidouble stranded deoxyribonucleic acid; Anti‐SS‐A, anti‐Sjögren's syndrome‐related antigen A; AST, aspartate aminotransferase; Ba, basophil; BNP, brain natriuretic peptide; BUN, blood urea nitrogen; Ca, calcium; Cl, chloride; CPK, creatine phosphokinase; Cr, creatinine; CRP, c‐reactive protein; Eo, eosinophil; Hb, hemoglobin; HbA1c, hemoglobin A1c; Ht, hematocrit; K, potassium; LDH, lactate dehydrogenase; Lym, lymphocyte; Mo, monocyte; Na, sodium; Neu, neutrophil; P, phosphorus; Plts, platelets; RBC, red blood cell; TP, total protein; UA, uric acid; WBC, white blood cell; γ‐GTP, γ‐glutamyltransferase.

**Table 2:** Endocrinal laboratory investigations

Endocrinal laboratory data		Laboratory investigations acquired during the hypoglycemic attack	
Neurohypophysis		Glucose	32 mg/dL
ADH	2.6 pg/mL	Insulin	7.7 µU/mL
Adenohypophysis		C‐Peptide	0.88 ng/mL
ACTH	10.8 pg/mL	Fajans index	0.24 (normal range < 0.3)
F	9.3 µg/dL	Tuner index	385 (normal range < 50)
TSH	1.060 µIU/mL		
fT3	2.09 pg/mL		
fT4	1.19 ng/dL		
PRL	22.0 ng/mL		
GH	0.52 ng/mL		
IGF‐1	47 ng/mL		
FSH	48.7 mIU/mL		
LH	24.1 mIU/mL		
E2	< 25 pg/mL		
Prog	0.2 ng/mL		

Abbreviations: ACTH, adrenocorticotropic hormone; ADH, antidiuretic hormone; E2, estradiol; F, cortisol; FSH, follicle‐stimulating hormone; fT3, free triiodothyronine; fT4, free thyroxine; GH, growth hormone; IGF‐1, insulin‐like growth factor‐1; LH, luteinizing hormone; PRL, prolactin; Prog, progesterone; TSH, thyroid‐stimulating hormone.

**Figure 3:**
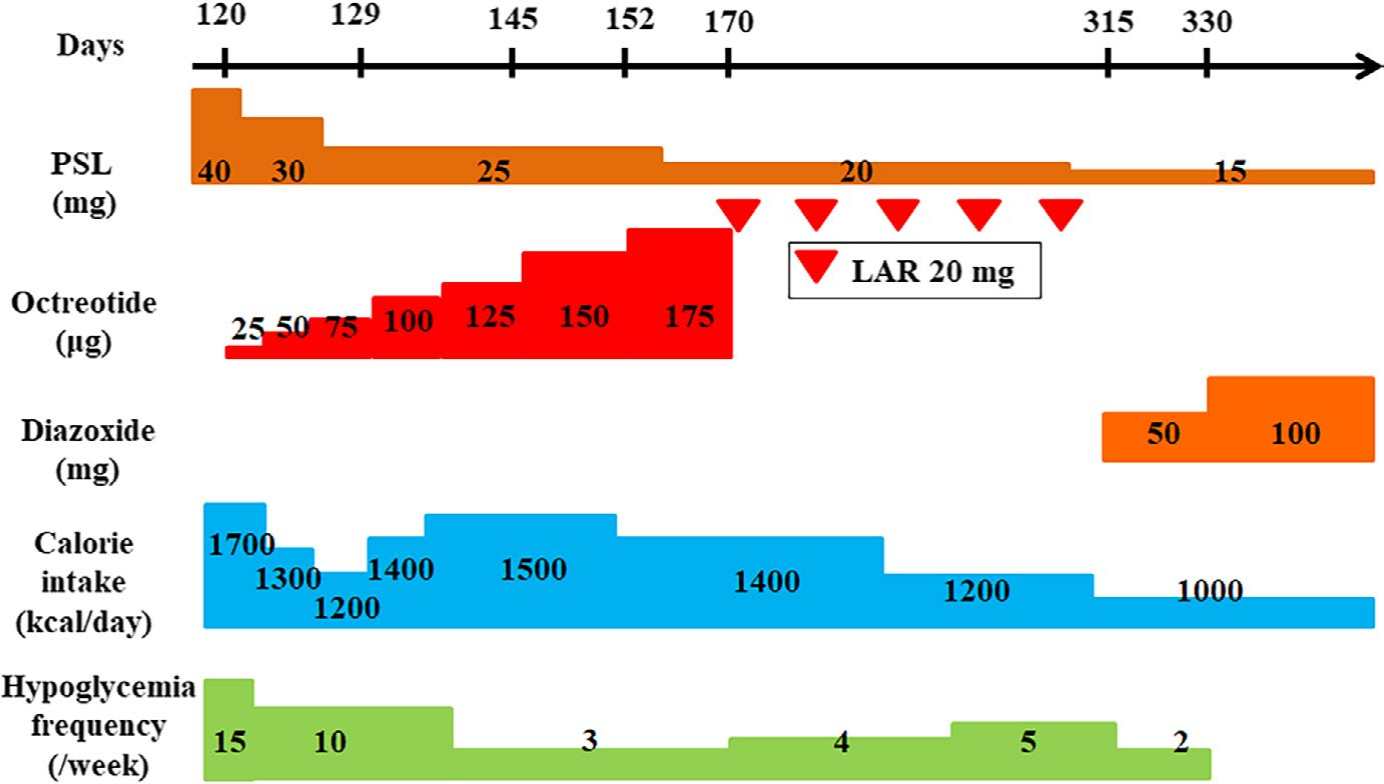
Clinical course of the patient. The figure shows the clinical progression of the patient over the duration of 7 months. After changing from the long‐acting release octreotide (LAR) to diazoxide, the frequency of hypoglycemic attacks decreased, and blood glucose levels became stable. Abbreviations: PSL, prednisolone; LAR, long‐acting release

She had valvular disease and low cardiac function, so we deemed surgical resection risky, and other nonsurgical ablative modalities were not available at our hospital, so we decided to start the patient on octreotide. As shown in Figure 3, octreotide was started from 25 μg daily dose and gradually increased the dose to 175 μg, and after approximately 2 months, it was switched to long‐acting release octreotide (LAR). However, since hypoglycemia was often observed during LAR treatment, the drug was considered ineffective for our patient, and we decided to switch to diazoxide. Although the patient had no apparent symptoms of heart failure, her cardiac function was low, and diazoxide could exacerbate her heart failure and weight loss (a body mass index of 13.8 due to malnutrition and wasting was seen). For these reasons, diazoxide was started at 50 mg/d (1.6 mg/kg), which is below the recommended starting dose of 3.0 mg/kg. The frequency of hypoglycemia decreased after diazoxide administration, and no side effects, such as heart failure or edema, were observed. After that, we determined the maintenance dose would be the recommended lower limit of 100 mg/d (3.2 mg/kg). The blood glucose transition was more stable with that small dose, as shown in the clinical course chart (Figure 3). Two months after the initiation of treatment with diazoxide, the laboratory data were not significantly different from the previous data (Table 3). She had no adverse side effects from diazoxide such as worsening of cytopenias and heart failure, and her blood glucose level was stable, so we continued treating her in the Internal Medicine outpatient division for 6 months. After that, our patient was transferred to the general hospital.

**Table 3:** Laboratory data 2 months after starting the treatment with diazoxide

Urinalysis		Blood chemistry	
Color	Yellow	CRP	1.79 mg/dL
pH	8.0	TP	5.5 g/dL
Protein	(‐)	Alb	2.7 g/dL
Occult blood	(‐)	AST	27 IU/L
Glucose	(‐)	ALT	18 IU/L
Ketone bodies	(±)	LDH	333 IU/L
		ALP	182 IU/L
		γ‐GTP	46 IU/L
Hematological investigations		CPK	60 IU/L
WBC	3900/µL	Na	133 mEq/L
Neu	73.0%	K	4.4 mEq/L
Lym	16.0%	Cl	96 mEq/L
Mo	10.0%	Ca	8.3 mg/dL
Eo	0.0%	P	3.6 mg/dL
Ba	0.0%	BUN	17 mg/dL
RBC	304 × 10^4^/µL	Cr	0.23 mg/dL
Hb	9.1 g/dL	UA	4.1 mg/dL
Ht	28.5%	glucose	105 mg/dL
Plts	15.9 × 10^4^/µL	BNP	217.3 pg/mL

Note

Abbreviations: Alb, albumin; ALP, alkaline phosphatase; ALT, alanine aminotransferase; AST, aspartate aminotransferase; Ba, basophil; BNP, brain natriuretic peptide; BUN, blood urea nitrogen; Ca, calcium; Cl, chloride; CPK, creatine phosphokinase; Cr, creatinine; CRP, c‐reactive protein; Eo, eosinophil; Hb, hemoglobin; Ht, hematocrit; K, potassium; LDH, lactate dehydrogenase; Lym, lymphocyte; Mo, monocyte; Na, sodium; Neu, neutrophil; P, phosphorus; Plts, platelets; RBC, red blood cell; TP, total protein; UA, uric acid; WBC, white blood cell; γ‐GTP, γ‐glutamyltransferase.

## 3 DISCUSSION

Surgery is considered the first line of treatment of insulinoma. However, because surgery was considered risky in this case, we decided to explore medical options. These medical options may include streptozotocin, everolimus, octreotide, and diazoxide, as described above. Streptozotocin is no longer used in the management of benign insulinoma due to it's nephrotoxicity and toxic effects on beta cells of the pancreas.^10^ Everolimus is also used as an antitumor agent, but susceptibility to infection is easily caused by immunosuppression.^11^ Due to the patient's steroid prescription, everolimus was considered suboptimal.

Therefore, our first choice was a somatostatin analog, due to the fact that it was easy to adjust the dose and because of the drug's hypoglycemia suppression and antitumor effects. Somatostatin analogs such as octreotide mainly act on somatostatin receptor 2 (SSTR2) and have the effect of suppressing hormone secretion from tumors, along with antitumor effects.^12^ However, the expression of SSTR2 in insulinoma is often unpredictable.^12^ It is also said that SSTR2 expression varies from case to case.^13^ In this case, the effect of the somatostatin analog was insufficient, and the expression of SSTR2 was probably low. For this reason, we chose diazoxide as the next treatment.

Diazoxide changes the transmembrane potential by opening the potassium (K) channel of pancreatic β cells. This closes the calcium (Ca) channel and lowers the intracellular Ca concentration to suppress insulin secretion.^6^ The recommended starting dose is 3‐5 mg/kg, and the maintenance dose is 3‐8 mg/kg divided into 2‐3 doses.^14^ Adverse effects include edema, hirsutism, thrombocytopenia, acute pancreatitis, etc may occur, and some of which have been reported in 47% of cases.^15^ Although there were few reports of diazoxide treatment, diazoxide was selected as the next treatment.

This was a rare case in which insulinoma and SLE were combined. To our knowledge, there are only a few reports of cases who had both SLE and insulinoma simultaneously.^16^ In SLE, steroid therapy often masks hypoglycemia which makes a diagnosis of insulinoma in such cases difficult. In fact, we first considered the cause of hypoglycemia to be due to a decrease in steroid dose or a side effect of the trimethoprim‐sulfamethoxazole combination. We suspected the presence of insulinoma because hypoglycemia was observed even when the steroid dose was stable and when the trimethoprim‐sulfamethoxazole combination was discontinued.

## 4 CONCLUSION

Pharmacological options are reserved for cases of benign insulinoma in which surgery is considered risky. Low‐dose Diazoxide can be used effectively to maintain a stable blood glucose level with minimal adverse effects in such cases.

## CONFLICT OF INTEREST

None declared.

## AUTHOR CONTRIBUTIONS

AY wrote the manuscript. TS reviewed the manuscript. NK added support for creating clinical data. TB was the neurosurgeon who participated in the creation of clinical course data. NS was the rheumatologist who participated in the creation of clinical course data. YK was responsible for drug research. MS KT and MO reviewed the manuscript.
